# Permeability and Stability of Hydrophobic Tubular Ceramic Membrane Contactor for CO_2_ Desorption from MEA Solution

**DOI:** 10.3390/membranes12010008

**Published:** 2021-12-22

**Authors:** Yunzhao Guo, Wenbo Qi, Kaiyun Fu, Xianfu Chen, Minghui Qiu, Yiqun Fan

**Affiliations:** State Key Laboratory of Materials-Oriented Chemical Engineering, College of Chemical Engineering, Nanjing Tech University, Nanjing 211816, China; yzguo@njtech.edu.cn (Y.G.); 202061204258@njtech.edu.cn (W.Q.); chenxianfu@njtech.edu.cn (X.C.); qiumh_1201@njtech.edu.cn (M.Q.); yiqunfan@njtech.edu.cn (Y.F.)

**Keywords:** membrane contactor, carbon dioxide, desorption, ceramic membrane

## Abstract

Ceramic membrane contactors hold great promise for CO_2_ desorption due to their high mass transfer area as well as the favorable characteristics of ceramic materials to resist harsh operating conditions. In this work, a hydrophobic tubular asymmetric alpha-alumina (α-Al_2_O_3_) membrane was prepared by grafting a hexadecyltrimethoxysilane ethanol solution. The hydrophobicity and permeability of the membrane were evaluated in terms of water contact angle and nitrogen (N_2_) flux. The hydrophobic membrane had a water contact angle of ~132° and N_2_ flux of 0.967 × 10^−5^ mol/(m^2^∙s∙Pa). CO_2_ desorption from the aqueous monoethanolamine (MEA) solution was conducted through the hydrophobic tubular ceramic membrane contactor. The effects of operating conditions, such as CO_2_ loading, liquid flow rate, liquid temperature and permeate side pressure, on CO_2_ desorption flux were investigated. Moreover, the stability of the membrane was evaluated after the immersion of the ceramic membrane in an MEA solution at 373 K for 30 days. It was found that the hydrophobic α-Al_2_O_3_ membrane had good stability for CO_2_ desorption from the MEA solution, resulting in a <10% reduction of N_2_ flux compared to the membrane without MEA immersion.

## 1. Introduction

Carbon dioxide (CO_2_) capture plays a key role in reducing CO_2_ emissions. Among current technologies for CO_2_ capture, amine scrubbing is considered to be the most well-established one, dominating industrial application in the short-to-medium terms [1]. However, the most pressing issue in this technology is the regeneration of solvent, which represents approximately two-thirds of operating cost [2]. Thus, any improvement in reducing energy usage, such as employing an advanced stripping configuration, will contribute to lowering capture costs [3].

Current challenges associated with the conventional CO_2_ desorption (or solvent regeneration) process at least include two most significant ones: (I) the liberation of free CO_2_ molecules from their compound form and (II) the recovery of useful heat from evaporated water vapor. Specifically, a process of CO_2_ desorption from amine solutions undergoes the decomposition of unstable carbamate and/or bicarbonate species into CO_2_ and amine molecules and then the release of CO_2_ molecules from the liquid phase to the gas phase. Accompanied by the CO_2_ desorption process, a large amount of water in a reboiler needs to be evaporated to act as a stripping vapor due to low equilibrium CO_2_ partial pressure. Typically, a reboiler is operated at a high temperature allowed by solvent stability or by the available steam supply. Elevated temperature does increase CO_2_ desorption flux. However, it requires high heat duty. Even though part of the vapor from the reboiler is cooled down to condense water in the stripper, the overhead vapor contains 1–5 mol of water vapor per mol of CO_2_, depending on reboiler temperature and solvent employed [4]. This situation will cause a massive loss of latent heat in the overhead condenser. If an advanced separator is developed to greatly increase the mass transfer area for CO_2_, reboiler temperature (or mass transfer driving force) could be significantly reduced.

Membrane contactors are potential candidates applied for CO_2_ desorption given their advantages of high specific surface area and high operational flexibility as well as easy modularization [5,6]. To date, much fewer studies regarding membrane contactors have been conducted for membrane CO_2_ desorption compared with membrane CO_2_ absorption. Overall, one of the key obstacles that cause this situation is that CO_2_ desorption is usually carried out at elevated temperatures, e.g., at 100–120 °C for elevated-pressure desorption or at 70–100 °C for vacuum desorption [7,8]. High temperature and chemical conditions require membrane materials to exhibit excellent characterizes. In the past decades, some polymeric membranes, most notably polyvinylidene fluoride (PVDF) [5], polytetrafluoroethylene (PTFE) [9] and polypropylene (PP) [10], have been used for CO_2_ desorption. Despite these polymeric materials exhibiting advantages of high specific surface area for mass transfer, in general, they underperform on anti-chemical degradation, anti-thermal aging and mechanical strength [6]. These drawbacks of polymeric membranes make them easily susceptible to undesired variations in membrane structure and properties, such as in morphology, microstructure, hydrophobicity, etc., and even to liquid leakage after long-term exposure to the evaluated-temperature chemical solution. Thus, the employment of other promising membrane materials that can withstand long-term harsh conditions is essential.

Tubular ceramic membranes have higher mechanical strength and chemical and thermal stabilities than polymeric membranes as well as hollow ceramic membranes under harsh operating conditions [11]. They have been applied for different harsh conditions such as membrane reaction [12,13], membrane distillation [14,15], membrane desorption [16], water heat recovery [17] and other applications [18,19]. They are probably more suitable than polymeric membranes for membrane CO_2_ desorption. However, the permeability and stability of tubular ceramic membranes used for CO_2_ desorption from amine solutions can be rarely found in the open literature.

Generally, the materials used for membrane desorption are hydrophobic. The hydrophobic surface enables the creation of a high liquid entry pressure (LEP) to avoid the entrance of feed solution into pores. Consequently, only CO_2_ and water vapor are able to pass through the hydrophobic pores. The pores filled with gas and vapor usually have higher mass transfer performance for CO_2_ compared with those filled with liquid since membrane desorption processes are driven by temperature and pressure differences. Moreover, the hydrophobic pores without wetting will improve thermal and chemical resistance for long-term performance [6]. Original ceramic materials are hydrophilic because of the presence of massive hydroxyl groups (–OH) on their surface and pores [20]. Recently, extensive studies have confirmed that ceramic membranes can be endowed with stable hydrophobicity by grafting hydrophobic groups, such as organosilane, on the membrane interface [21]. Advances in hydrophobic modification increase the opportunities for the industrial application of ceramic membranes for CO_2_ desorption. In this work, hexadecyltrimethoxysilane (C16) ethanol solution was used for hydrophobic modification. The reasons for that are presented as follows. First, C16 is cheap, easy to store and less toxic compared to some commonly used modifiers, such as fluoroalkylsilanes (FAS). In addition, ethanol is a harmless and non-toxic solvent, can be considered as an environmentally friendly alternative to traditional grafting solvents such as acetone harmful solvents during the grafting process. Furthermore, the C16 ethanol solution had been used for the fabrication of hydrophobic zirconia (ZrO_2_) and alumina (Al_2_O_3_) membranes. The grafted ceramic membranes possessed high hydrophobicity and performed well in the processes of membrane absorption for gas separation [21], water–oil separation [22] and membrane distillation for desalination [14].

In this work, a hydrophobic tubular asymmetric alpha-alumina (α-Al_2_O_3_) ceramic membrane contactor for CO_2_ desorption from an aqueous monoethanolamine (MEA) solution was investigated in terms of mass transfer performance and stability. The mass transfer performance of the hydrophobic asymmetric ceramic membrane was experimentally evaluated in terms of the N_2_ flux and, more importantly, CO_2_ desorption flux under various conditions, including temperature, pressure and liquid flow rate. In addition, the stability of the original and hydrophobic membranes was evaluated in terms of the N_2_ flux, water contact angle and morphology before and after the immersion of the ceramic membrane in aqueous MEA solution at 373 K for 30 days.

## 2. Experiment

### 2.1. Materials

The ceramic membrane, which was fabricated by coating an α-Al_2_O_3_ membrane layer on the internal surface of tubular α-Al_2_O_3_ support, was supplied by Membrane Industrial Park, (Jiangsu, China). Reagent grade MEA with a purity of ≥99% was purchased from Shanghai Ling Feng Chemical Reagent Co., Ltd. (Shanghai, China). Commercial grade N_2_ and CO_2_ were supplied by Nanjing Ning Wei Medical Oxygen, Co., Ltd., Nanjing, China. Reagent grade hexadecyltrimethoxysilane (C16) with a purity of ≥85% (GC) was purchased from Shanghai Aladdin Chemical Reagent Co. Ltd., (Shanghai, China).

### 2.2. Preparation and Characterization of the Hydrophobic Membrane

**Figure 1:**
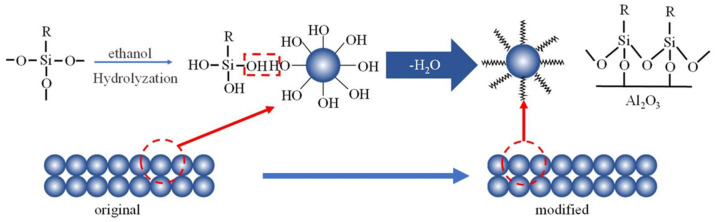
Schematic diagram of the hydrophobically modification process.

The surface modifier was prepared by mixing the concentrated C16 with ethanol and a certain amount of nitric acid (about 3 mL 0.1 mol/L HNO_3_ per 1 L solution) to 0.1 mol/L C16 at room temperature for 24 h. The raw tubular membranes were dried and immersed into the modifier solution at 30 °C for 12 h. In the modification process, the –OCH_3_ group in silane molecule undergoes hydrolysis reaction to form silanol (R–Si–(OH)_3_) to possess hydrophobicity (Figure 1). The modified membranes were taken out and rinsed with deionized water and then dried at 110 °C for 6 h for curing the silane-modified silica to improve the stability of the hydrophobic membrane. Roughly, 1 L modified solution can be used for 5 membrane tubes. The membranes were stored at room temperature. The properties of the tubular asymmetric α-Al_2_O_3_ membrane to be characterized include water contact angle, gas permeation and morphology. The water contact angle of the ceramic membranes was measured by a contact angle analyzer (Dataphysics-OCA20, DataPhysics Instruments GmbH Co., Ltd., Filderstadt, Germany). The porosity of the membrane was characterized by an ellipsometry device (Complete EASEM-2000U, J.A. Woolam, Lincoln, NE, USA). The tests of gas permeation were carried out to investigate the effect of hydrophobic modification on membrane microstructure. Pure N_2_ was used to investigate the gas permeation. The test module containing a ceramic membrane with 11 cm length was prepared to determine the N_2_ permeance of the membrane. The upstream pressure was increased at 0.05 MPa intervals up to 0.4 MPa. The N_2_ was fed into the lumen side of the module, and the permeation rates were measured at 25 °C in the shell side using a rotor flow meter. The morphology of the membrane was assessed using field emission scanning electron microscopy (FESEM, S-4800, Hitachi High-Tech, Tokyo, Japan).

The N_2_ permeance flux can be calculated as follows:

(1) $$J_{N_{2}}=\frac{G}{V_{m}\times A\times \Delta P}\times \frac{T}{273.15}$$

where *J*_N2_ is the N_2_ permeance flux, mol∙m^−2^∙s^−1^∙Pa^−1^; G is the volume flow rate of N_2_ from the permeation side, L/s; *V*_m_ is the gas molar volume, 22.4 L/mol; A is the area of the membrane layer, m^2^; Δ*P* is the transmembrane pressure difference, Pa; T is the temperature, K.

### 2.3. Sample Analysis

The solutions were prepared by mixing concentrated MEA with deionized water to desired concentrations. The MEA concentration was verified by titration against 1.0 mol/L hydrochloric acid (HCl) using methyl orange as an indicator. The liquid phase CO_2_ loading was determined in a Chittick apparatus by the standard method presented by the Association of Official Analytical Chemists (AOAC) apparatus [23]. The CO_2_ concentration in the gas phase was determined by a CO_2_ analyzer (COZIR^TM^ Wide Range, CO_2_ Meter, Ormond Beach, FL, USA). A gas flow totalizer (D07-19B, Beijing Sevenstar Electronics Co., Ltd., Beijing, China) was used to measure the accumulated flow rate of the stripping CO_2_.

### 2.4. Experimental Apparatus and Procedure for Membrane CO_2_ Desorption

**Figure 2:**
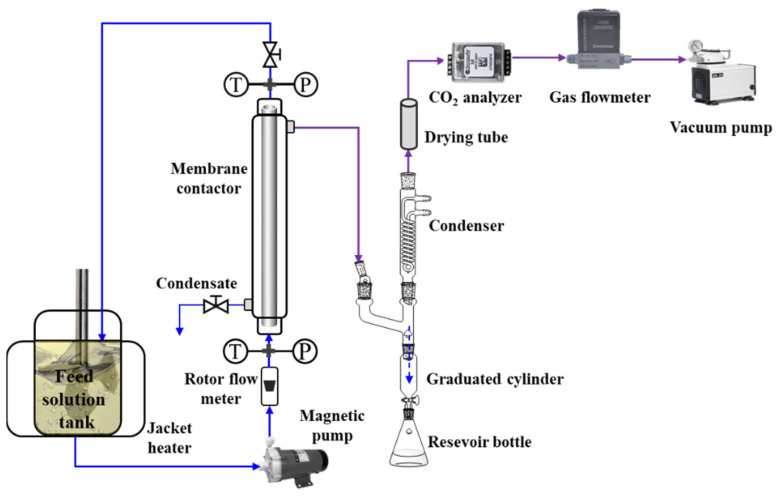
Schematic diagram of the membrane CO_2_ stripping process.

The schematic diagram of the experimental setup for CO_2_ desorption is shown in Figure 2. A hydrophobic tubular asymmetric α-Al_2_O_3_ membrane was encapsulated in a 304 stainless module to form the membrane contactor. The characteristics of the membrane contactor are presented in Table 1. The CO_2_-rich aqueous MEA solution in a heating tank was continuously pumped into the lumen side of the membrane contactor and then recycled back to the tank. The liquid flow rate was controlled by a rotameter (accuracy: ±2%). The temperatures and pressures of the solvent at the inlet and outlet of the membrane contactor were monitored using PT100-type thermal sensors (0−200 °C) and SIN-P300 pressure transmitters (0−0.6 MPa), respectively. In addition, the reduced pressure of the permeable side of the membrane contactor is generated by a vacuum pump and is determined by a pressure transmitter (−0.1–0 MPa). The vaporized H_2_O was extracted from the membrane contactor and then was condensed. The condensate was determined by a precise graduated cylinder. The stripped CO_2_ was online measured by a gas flow totalizer (D07-19B, Beijing Sevenstar Electronics Co. Ltd., Beijing, China), which enables converting it into the standard state by automatic temperature calibration and connecting to a computer to collect instantaneous and cumulative flow rates once per second.

The CO_2_ permeancec flux can be calculated as follows:

(2) $$J_{CO_{2}}=\frac{F}{V_{m}\times A}$$

where *J*_CO__2_ is the CO_2_ permeance flux, mol∙m^−2^∙s^−1^; *F* is the flow rate measured by mass flowmeter, L/s.

### 2.5. Stability Study of the α-Al_2_O_3_ Membrane

**Figure 3:**
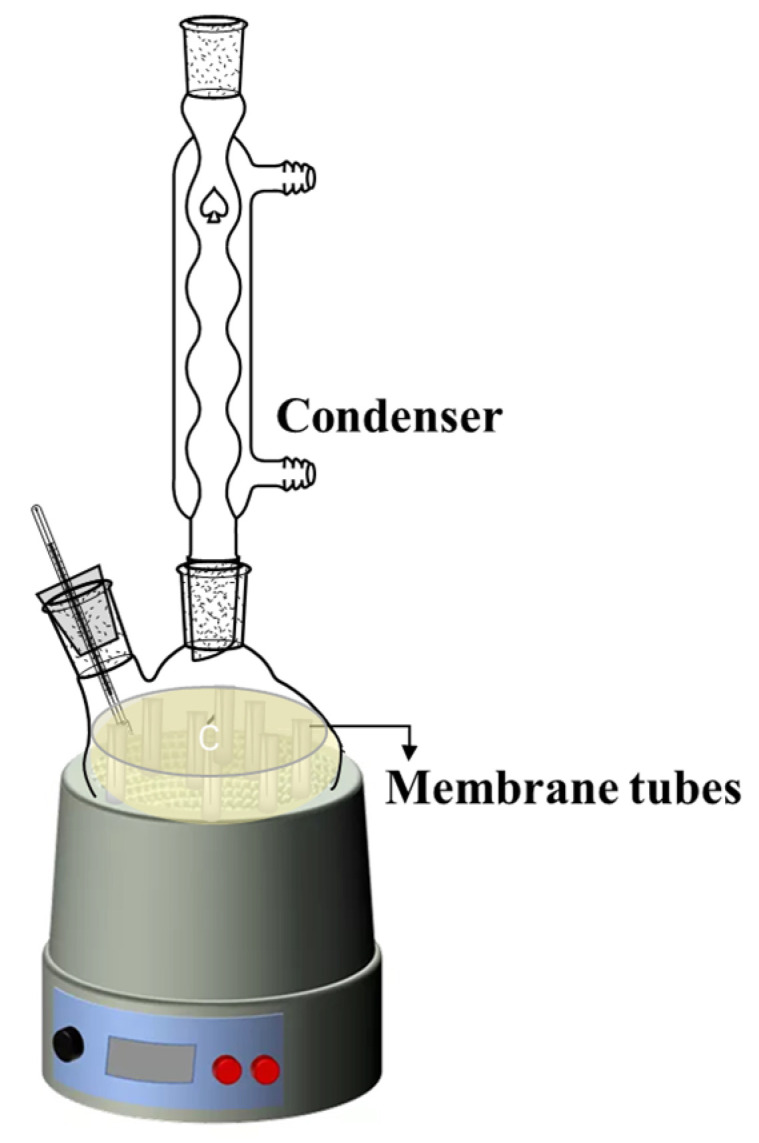
Schematic diagram of stability test.

The thermal and chemical stability of the membranes was studied as follows: the asymmetric α-Al_2_O_3_ membranes were immersed in a 5.0 mol/L MEA solution at 373 K for 30 days, as shown in Figure 3. After the 30 days of immersion, the membranes were taken out and washed with distilled water, then dried at room temperature. Then, the membrane samples were studied via FESEM analysis and gas permeation.

## 3. Results and Discussion

### 3.1. Characterization Results of the Hydrophobic Ceramic Membrane

**Figure 4:**
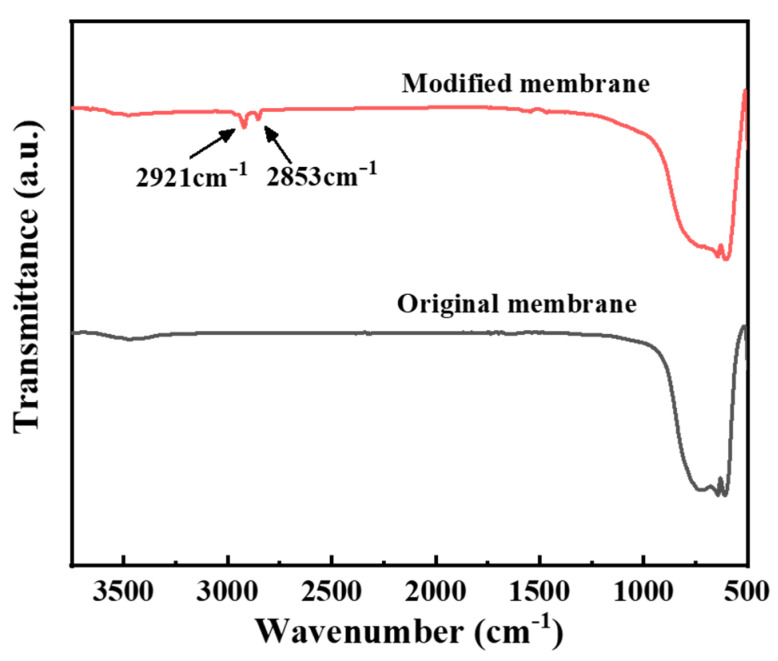
Fourier transform infrared spectrum (FTIR) of the α-Al_2_O_3_ membrane before and after modification.

**Figure 5:**
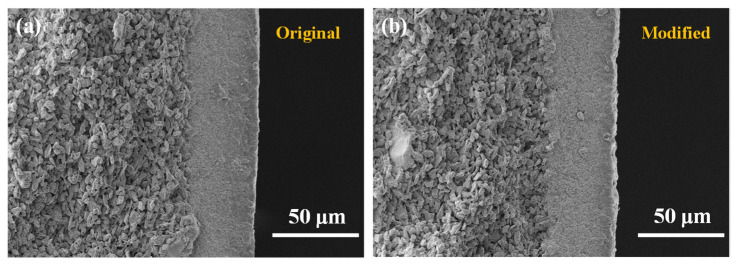
Scanning electron microscope (SEM) images of the α-Al_2_O_3_ membrane (**a**) before and (**b**) after modification.

**Figure 6:**
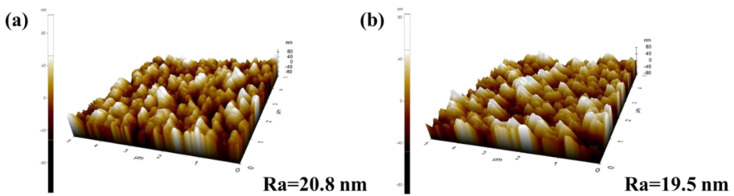
Atomic force microscope (AFM) images of the α-Al_2_O_3_ membrane (**a**) before and (**b**) after modification.

To characterize the hydrophobic membrane, Fourier transform infrared spectrum (FTIR) determination was firstly conducted to see the bond variation. It can be observed in Figure 4 that the asymmetric stretching vibration peaks and symmetric stretching vibration peaks of –CH_2_– appeared at 2921 cm^−1^ and 2853 cm^−1^ on the modified spectrum, indicating that the silane molecules have been successfully grafted to the surface of the ceramic membrane. Subsequently, the cross-sectional and surface roughnesses of the original membrane and the modified membrane are determined via FESEM and AFM, as presented in Figure 5 and Figure 6, respectively. No obvious change can be observed from the FESEM and AFM images of the membranes before and after hydrophobic modification, indicating that the effect of the hydrophobic modification using C16 on membrane microstructure was insignificant.

**Figure 7:**
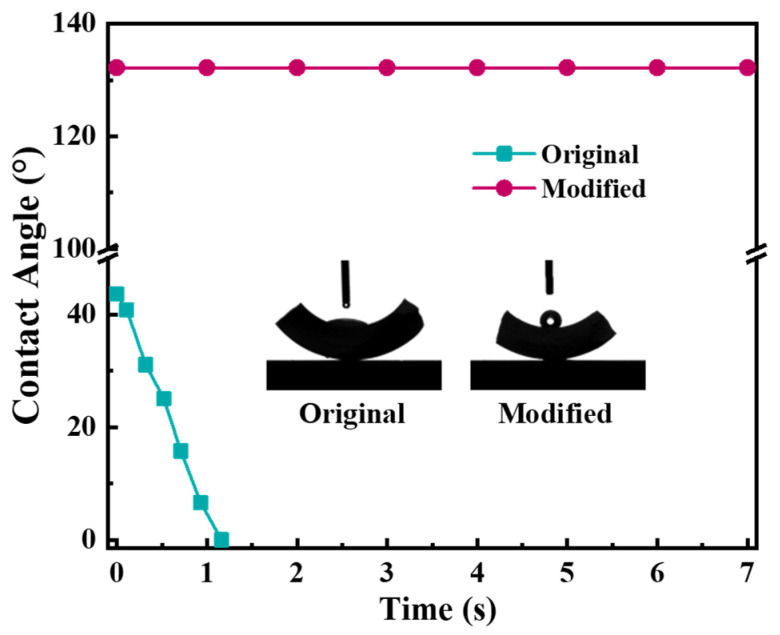
The water contact angle of the ceramic membrane before and after modification.

The hydrophobicity of the grafted ceramic membrane was tested in terms of water contact angle. The water contact angle of the original membrane decreased sharply from 40° to 0° in a few seconds due to the presence of hydroxyl groups (−OH) on the membrane surface, as shown in Figure 7. By contrast, the contact angle of the grafted membrane kept stably greater than 130°, indicating the modifier had been satisfactorily grafted and the surface of the ceramic membrane was hydrophobic.

**Figure 8:**
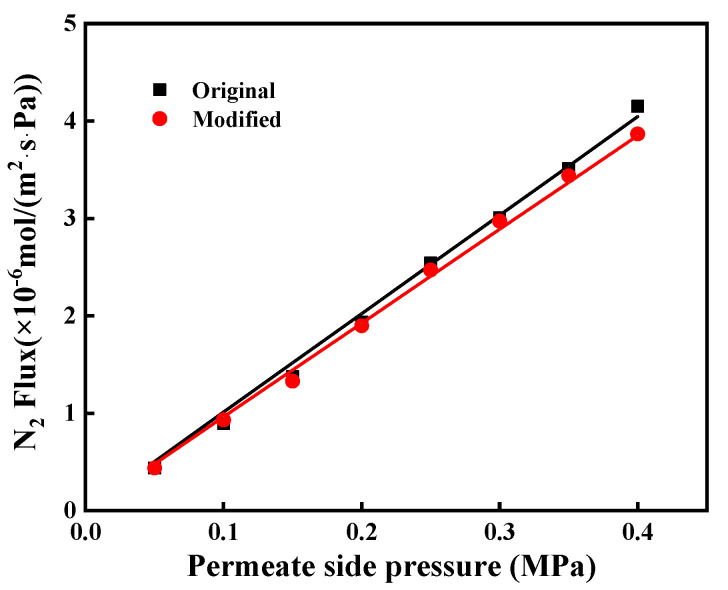
The N_2_ flux of the ceramic membrane before and after modification.

The N_2_ permeances of the original and grafted ceramic membranes at the transmembrane pressure of N_2_ ranging from 0.05 to 0.40 MPa, with the highest permeation fluxes of 1.01 × 10^−5^ and 0.967 × 10^−5^ mol/(m^2^∙s∙Pa), respectively, as shown in Figure 8. It indicates that the grafted ceramic membranes exhibited high hydrophobicity concurrently without causing much reduction of gas permeation. It was likely due to that the grafted C16 layer on the inner surface of pore channels was very thin. The thickness of the grafted layer was only a few nanometers (<3 nm) [24,25], which was much smaller than the pore sizes (0.1 μm for the top layer and 1.0 μm for the support layer). Therefore, the hydrophobic modification had an insignificant effect on the gas permeation.

### 3.2. Effects of Key Operating Conditions 

Operating conditions are important to CO_2_ desorption performance. To investigate the effects of several key operating parameters on the membrane CO_2_ desorption performance, experiments were conducted at an MEA concentration of 5.0 mol/L, liquid temperature ranging from 363.15 to 373.15 K, CO_2_ loading ranging from 0.2 to 0.45 mol CO_2_/mol MEA, liquid flow ranging from 200 to 400 mL/min and permeate pressure ranging from 50 to 80 kPa. 

**Figure 9:**
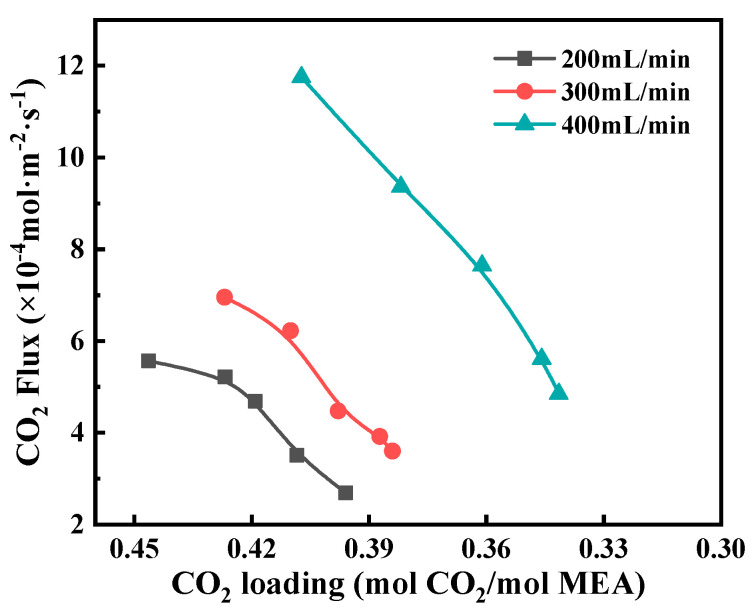
The effect of CO_2_ loading on CO_2_ flux at different liquid flow rates. Experimental conditions: *T* = 373 K, *P* = 60 kPa, *C*_MEA_ = 5.0 mol/L.

The effect of CO_2_ loading on the CO_2_ stripping flux can be seen in Figure 9. With the decrease of CO_2_ loading, the CO_2_ stripping flux decreased significantly. This is because the decrease in CO_2_ loading would lower the CO_2_ equilibrium partial pressure, reflecting the smaller driving force for CO_2_ mass transfer. Meanwhile, an increase in liquid flow was of great benefit to improving the CO_2_ stripping flux. This is because as the liquid velocity increased, the liquid temperature and CO_2_ loading were little changed and maintained at high levels, thus keeping high mass transfer performance. In addition, an increase in the liquid flow resulted in reduced liquid phase mass transfer resistance which had a great effect on the overall mass transfer resistance. It should be noted that a high liquid flow means a fast circulation rate for liquid solution circulating between the membrane contactor and the reboiler, which will consume more pump energy. Therefore, it is important to choose an optimized liquid flow.

**Figure 10:**
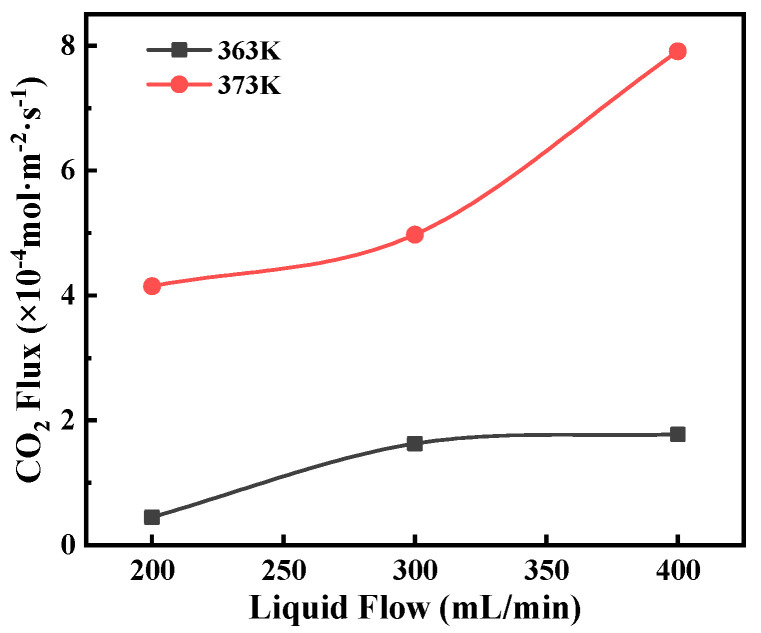
The effect of feed flow rate on CO_2_ flux at different temperatures. Experimental conditions: P = 60 kPa, *C*_MEA_ = 5.0 mol/L, CO_2_ loading = 0.45 mol CO_2_/mol MEA.

An increase in liquid temperature was of great benefit to increasing the CO_2_ stripping flux, as shown in Figure 10. This is because temperature directly affects CO_2_ equilibrium solubility and diffusion coefficients. The CO_2_ solubility in the MEA solution decreases exponentially with temperature [26], and the diffusivity increases in multiples of 4 by increasing the temperature by 10 K [27,28]. Thus, an increase in operating temperature leads to increases in both driving force and mass transfer coefficient for CO_2_ stripping. Moreover, an increase in the feed flow rate enabled reducing the temperature difference between the liquid bulk and the liquid–membrane interface, resulting in increased transmembrane pressure difference. The 363 K curve tended to a maximum as the feed flow rate further increased. This might be because the mass transfer in the feed side was negligible, and the transport in pore sizes governed the overall mass transfer process. 

**Figure 11:**
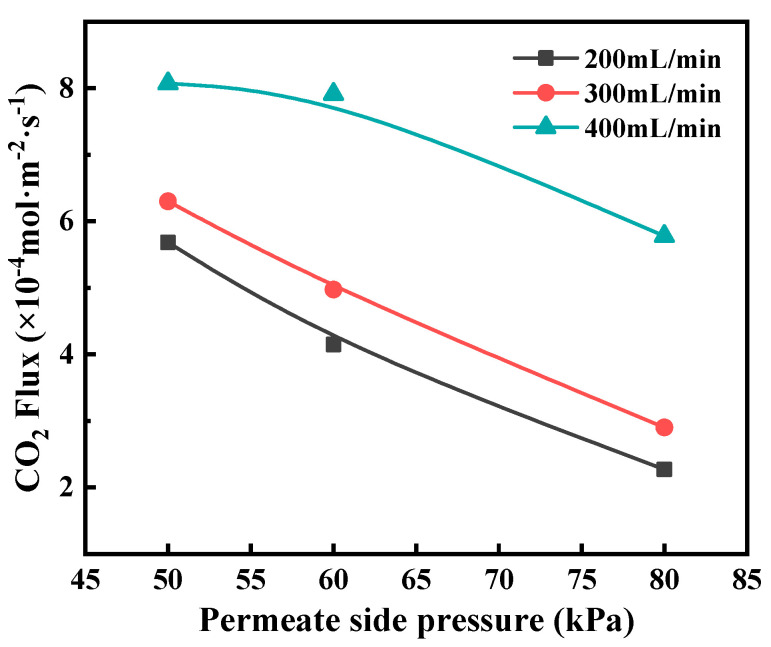
The effect of permeate pressure on CO_2_ flux at different liquid flow rate. Experimental conditions: *T* = 373 K, *C*_MEA_ = 5.0 mol/L, CO_2_ loading = 0.45 mol CO_2_/mol monoethanolamine (MEA).

Regeneration pressure also impacts CO_2_ desorption flux. Lowering permeate side pressure enhanced the CO_2_ desorption flux, as shown in Figure 11. It can be explained that the decrease in the permeate side pressure is favorable for decreasing the CO_2_ partial pressure in the gas phase, thus improving the CO_2_ stripping driving force. However, a too low permeate side pressure will lead to the considerably high energy consumption of the vacuum pump. A moderate degree of vacuum condition is in favor of improving the CO_2_ membrane stripping performance, facilitating the CO_2_ transport in the permeate side, concurrently will not cost too much energy.

Compared to other studies reported on membrane contactors for CO_2_ desorption using MEA solution, the hydrophobic tubular asymmetric α-Al_2_O_3_ membrane exhibited competitive mass transfer performance, as shown in Table 2.

### 3.3. The Stability of the Modified Ceramic Membrane

In industrial applications, membrane stability is an important issue in the membrane process for CO_2_ desorption from amine solutions. It determines how long a membrane can be operated. Therefore, not only permeate flux but also the thermal and chemical stability is critical for a membrane to be employed in CO_2_ desorption. Here, the hydrophobically modified α-Al_2_O_3_ membrane after 30 days’ immersion in MEA solution was characterized and compared with that without immersion.

**Figure 12:**
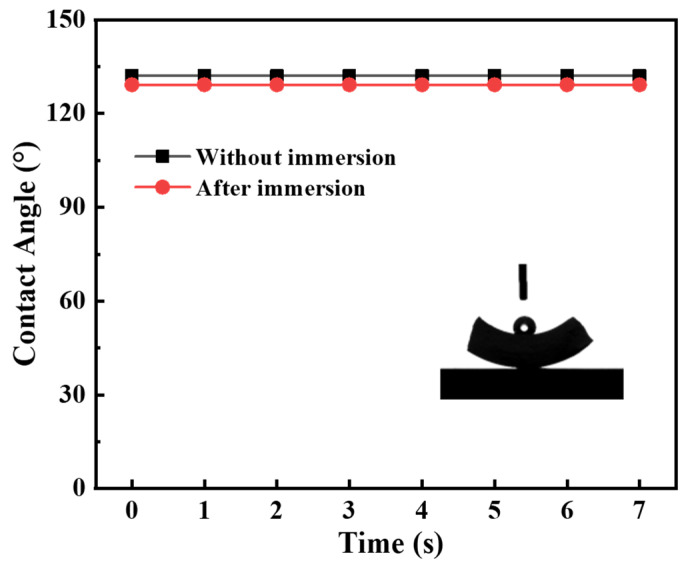
The water contact angle of the ceramic membrane after 30 day of immersion.

**Figure 13:**
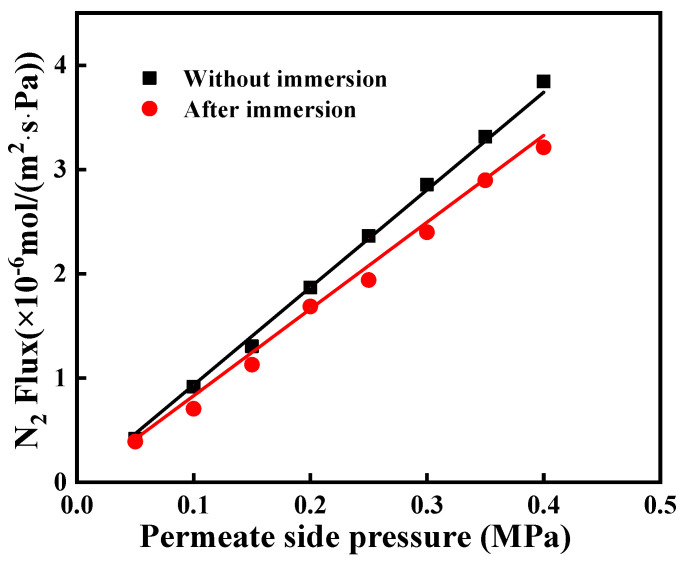
The N_2_ flux of the ceramic membrane after 30 day of immersion.

In this work, the contact angle, gas permeance and morphology of the immersed α-Al_2_O_3_ membrane were evaluated in order to investigate its thermal and chemical stability. As shown in Figure 12, the water contact angle of the immersed membrane was very close to that of the membrane without immersion. It means that the immersed membrane maintained good hydrophobicity. In addition, the gas permeation of immersed membrane the performance of unimmersed membrane at the transmembrane pressure of N_2_ ranging from 0.05 to 0.40 MPa, as shown in Figure 13. They had permeation fluxes of 0.932 × 10^−5^ and 0.847 × 10^−5^ mol/(m^2^∙s∙Pa), respectively. These results indicate that the MEA solution has a small effect on the stability of the modified membrane; however, the effect was acceptable.

**Figure 14:**
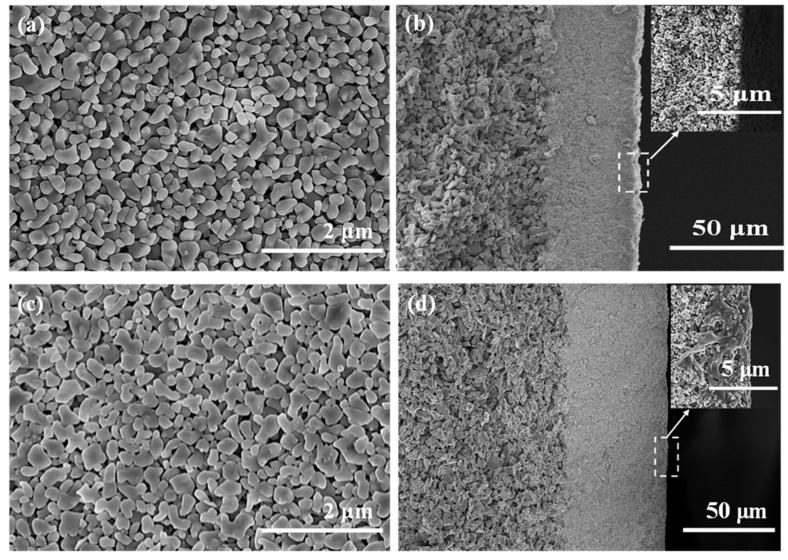
Field emission scanning electron microscope (FESEM) images of the α-Al_2_O_3_ membrane before and after immersion in MEA solution. (**a**) surface and (**b**) cross-section of the unimmersed membrane; (**c**) surface and (**d**) cross-section of the immersed membrane.

The microstructure of the hydrophobic membranes before and after immersion was presented in Figure 14 to observe the effect of MEA solution on the stability of the modified membrane. As shown in Figure 14a,c, no obvious variation can be found between the surface morphology of the hydrophobic ceramic membrane before and after the immersion in MEA solution. From the cross-sectional FESEM images, it can be found that the near-surface of the ceramic membrane was partially corroded after immersion in MEA solution at 100 °C for 30 days, which explained why the N_2_ flux of the immersed membrane showed a little decrease. In the membrane desorption process, a hydrophobic membrane can prevent the permeation of reactive MEA into pores; thus, it is an effective way to reduce corrosion.

## 4. Conclusions

A hydrophobic ceramic membrane was fabricated via grafting a hexadecyltrimethoxysilane ethanol solution and tested in terms of water contact angle, pure N_2_ permeability and CO_2_ desorption performance. The results showed that the modification strategy enables the grafted ceramic membranes to exhibit hydrophobicity higher than 130° concurrently without causing much reduction of gas permeability (less than 5%) compared to the original membrane without modification. CO_2_ desorption from MEA solution was conducted through the tubular asymmetric membrane. The results demonstrated that the CO_2_ loading, liquid flow rate, liquid temperature and permeate pressure were the key parameters on the CO_2_ desorption flux. The CO_2_ flux was found to be 1.17 × 10^−3^ (mol·m^−2^·s^−1^) at feed temperature of 373 K, permeate side pressure of 60 kPa, MEA concentration of 5.0 mol/L, CO_2_ loading of 0.41, feed flow rate of 400 mL/min. Moreover, stability tests of immersing the membrane into a 5.0 mol/L aqueous MEA solution at 373 K for 30 days were also performed to investigate the stability of the hydrophobic α-Al_2_O_3_ membrane. The experimental results showed that the MEA solution did affect the membrane stability, however, was acceptable (less than 10%). 

## Figures and Tables

**Table 1:** Characteristics of the membrane contactor.

Membrane Properties	Values	
	Membrane Layer	Support Layer
Mean pore size	0.1 (μm)	1.0 (μm)
Thickness	40 (μm)	2.0 (mm)
Porosity	0.4	0.4
Tortuosity factor	2.5	2.5
Membrane tube (OD/ID)	12/8 (mm)	
Module (ID)	22 (mm)	
Length	600 (mm)	

Note: OD and ID are outer and inner diameters, respectively.

**Table 2:** Performance of several kinds of membranes for CO_2_ desorption from MEA solution.

Material	CO_2_ Flux (mol·m^−2^·s^−1^)	Absorbent Concentration (mol/L)	CO_2_-Loading (mol CO_2_/mol Absorbent)	Feed Temperature (K)	Permeate Side Pressure (kPa)	Ref.
PVDF	3 × 10^−4^	5.0	0.45	373	100	[29]
PDMS + Psf	1 × 10^−3^	5.0	0.49	373	120	[30]
PTFE	5 × 10^−4^	5.0	0.45	373	100	[9]
Al_2_O_3_	2.56 × 10^−3^	5.0	0.45	353	61	[31]
PP	2.2 × 10^−4^	3.4	0.53	343	52	[32]
α-Al_2_O_3_	1.17 × 10^−3^	5.0	0.41	373	60	This work

## Data Availability

All the data supporting the findings of this study are available within the article.
